# Alterations of structure and functional connectivity of visual brain network in patients with freezing of gait in Parkinson’s disease

**DOI:** 10.3389/fnagi.2022.978976

**Published:** 2022-09-07

**Authors:** Lu Gan, Rui Yan, Dongning Su, Zhu Liu, Guozhen Miao, Zhan Wang, Xuemei Wang, Huizi Ma, Yutong Bai, Junhong Zhou, Tao Feng

**Affiliations:** ^1^Department of Radiology, Beijing Tiantan Hospital, Capital Medical University, Beijing, China; ^2^Center for Movement Disorders, Department of Neurology, Beijing Tiantan Hospital, Capital Medical University, Beijing, China; ^3^China National Clinical Research Center for Neurological Diseases, Beijing, China; ^4^Maranatha High School, Pasadena, CA, United States; ^5^Department of Neurosurgery, Beijing Tiantan Hospital, Capital Medical University, Beijing, China; ^6^Hinda and Arthur Marcus Institute for Aging Research, Hebrew SeniorLife, Roslindale, MA, United States; ^7^Harvard Medical School, Boston, MA, United States; ^8^Parkinson’s Disease Center, Beijing Institute for Brain Disorders, Capital Medical University, Beijing, China

**Keywords:** Parkinson’s disease, freezing of gait, magnetic resonance imaging, visual cortex, movement disorders

## Abstract

Freezing of gait (FOG) is a disabling gait disorder common in advanced stage of Parkinson’s disease (PD). The gait performance of PD-FOG patients is closely linked with visual processing. Here, we aimed to investigate the structural and functional change of visual network in PD-FOG patients. Seventy-eight PD patients (25 with FOG, 53 without FOG) and 29 healthy controls (HCs) were included. All the participants underwent structural 3D T1-weighted magnetic resonance imaging (MRI) and resting state functional MRI scan. Our results demonstrated a significant decrease of right superior occipital gyrus gray matter density in PD-FOG relative to non-FOG (NFOG) patients and healthy controls (PD-FOG vs. PD-NFOG: 0.33 ± 0.04 vs. 0.37 ± 0.05, *p* = 0.005; PD-FOG vs. HC: 0.37 ± 0.05 vs. 0.39 ± 0.06, *p* = 0.002). Functional MRI revealed a significant decrease of connectivity between right superior occipital gyrus and right paracentral lobule in PD-FOG compared to PD-NFOG (*p* = 0.045). In addition, the connectivity strength was positively correlated with gray matter density of right superior occipital gyrus (*r* = 0.471, *p* = 0.027) and negatively associated with freezing of gait questionnaire (FOGQ) score (*r* = -0.562, *p* = 0.004). Our study suggests that the structural and functional impairment of visual-motor network might underlie the neural mechanism of FOG in PD.

## Introduction

Freezing of gait (FOG) is one kind of severe gait disorder that is commonly presented in mid- and late-stage of Parkinson’s disease (PD) (Perez-Lloret et al., 2014). Patients with FOG in PD (i.e., PD-FOG) often have episodic feeling that their feet “glue” on the floor despite the motive of walking forward. It often occurs during gait initiation, turning around and/or termination of walking, leading to increased risk of falling and fracture, disability, loss of functional independence and worsening of life quality in people with PD-FOG (Nonnekes et al., 2015). Therefore, it is critical to look into the insights into the pathology of FOG in PD, and to characterize the potential contributors to this serious movement disorder. The knowledge obtained can thus ultimately help the optimization of the therapeutic, rehabilitative, and management strategies for people suffering from PD-FOG.

The regulation of gait is dependent upon multiple biophysiological components, including visual, proprioceptive, and vestibular information (Takakusaki, 2013). Among them, visual inputs provide critical information on the environment to help the formation of appropriate locomotion when walking. Studies have shown that altered vision is related to the disrupted gait regulation in people with PD-FOG. For example, Vanegas-Arroyave et al. (2022) observed that the sight in people with PD-FOG was limited to a narrow area of the ground rather than to the destination while walking, indicating increased dependence on visual feedback from proximal areas (Vanegas-Arroyave et al., 2022). Besides, the modulation of visual cues is associated with the presence of FOG. For example, Mestre et al. (1992) observed that the gait block could be avoided when cutting off the visual cue by closing eyes. They considered that hypersensitivity to visual stimulation provoked FOG and keeping away from visual cues led to alleviation. The gait performance of PD-FOG can be greatly affected by visual cues. For example, it was observed that PD-FOG can be unfrozen and start to walk by simply drawing a transverse line on the floor in front of them (Naismith et al., 2010). On the contrary, using stroboscopic light of stripes can lead to notable increased FOG episodes during walking (Azulay et al., 2006). These observations suggest that the altered perception and processing of visual information play important roles in FOG.

The perception and processing of visual information rely on distributed brain cortical network. The visual network primarily locates in the occipital lobe and ventral temporal cortices. Several studies have shown functional changes (e.g., hypoactivation) in this network in those with FOG. For example, resting state functional magnetic resonance imaging (fMRI) analyses revealed decline of connection inside the right occipitotemporal gyrus (Tessitore et al., 2012) and the left inferior occipital gyrus (Canu et al., 2015) within the visual network (VN). On the contrary, FOG alleviation after subthalamic nucleus deep brain stimulation (STN-DBS) was detected to be coupled with higher metabolism in parieto-temporo-occipital areas (Lyoo et al., 2007). In walking or turning virtual reality (VR) tasks, PD-FOG showed increased recruitment of visual cortex (Gilat et al., 2015) and higher blood oxygenation level-dependent (BOLD) response in extra-striate visual cortex (Shine et al., 2013). Most of these studies focused on the activation within VN. Yu et al. (2021) found that the resting-state connectivity between the sensory-motor network and medial visual network was decreased. However, the exact brain area of visual network was not specified. The interaction between specific regions in VN and other PD-related motor regions in resting-state needs to be further characterized. Additionally, little is known to how the structural changes within the regions of VN that are associated with PD-FOG, and the relationships between the structural alterations, functional changes within this network and the severity of FOG have not been elucidated.

Here in this study, we aimed to first identify the regions of VN with significant structural differences in PD-FOG as compared to PD-NFOG and HCs, by measuring the gray matter density (GMD), an important characteristic that has been associated with gray matter atrophy (Ashburner and Friston, 2000; Dalrymple et al., 2021). We then characterized the resting-state functional connectivity (FC) of BOLD signals between those identified regions with structural difference and the regions that were previously reported to be pertaining to FOG. After the characterization of GMD and resting-state FC in FOG, we examined the relationship between these characteristics of VN and the severity of FOG. We hypothesized that those with greater structural and functional alterations of VN would have greater severity of FOG.

## Materials and methods

### Subject inclusion

A total of 78 patients with PD were included, of which 53 were PD-NFOG and 25 were PD-FOG patients, and 29 healthy controls were also included. The diagnosis of PD was based on the United Kingdom Parkinson’s Disease Society Brain Bank criteria for PD (Hughes et al., 1992). And the diagnosis was confirmed by 2 experienced neurologists. PD-NFOG subjects were rated stage 2–4 of the Hoehn and Yahr (H-Y) staging scale. PD-FOG patients were rated stage 2.5–4. PD-FOG were identified based on following criteria: (a) score ≥ 1 on item 3 of the Freezing of Gait Questionnaire (FOGQ) (“do you feel that your feet get glued to the floor while walking, making a turn or when trying to initiate walking?”) (Giladi et al., 2009; Wu et al., 2020); (b) score ≥ 1 on item 14 of Unified Parkinson’s Disease Rating Scale part II (UPDRS II), which confirmed the patients have the feeling that their feet were glued to the floor when starting to walk or turning (Knobl et al., 2012; Perez-Lloret et al., 2014); (c) recognition of typical FOG by experienced neurologists. Patients were classified as PD-FOG when they met all the criteria above. PD-NFOG patients were those who never experienced FOG and scored zero on the questions. Patients with any one of the following conditions were excluded: (a) visual impairment or ophthalmic diseases, (b) severe tremor or dyskinesia, (c) Mini Mental State Exam (MMSE) score < 24, (d) musculoskeletal or orthopedic diseases that impacted gait, (e) contraindications to MRI such as metal devices in the body or claustrophobia. The healthy controls were recruited from local individuals without neurological or psychiatric disorders.

The study was approved by the Ethics Committee of Beijing Tiantan Hospital affiliated to Capital Medical University. All subjects gave written informed consent according to the Helsinki Declaration on ethical principles for medical research. Study procedures were provided and explained to each of the subjects in person at least 1 week before the assessment.

### Clinical assessments

Motor symptoms of PD patients were screened by H-Y Staging Scale and motor section of the Unified Parkinson’s Disease Rating Scale (UPDRS-III). The MMSE and Montreal Cognitive Assessment (MoCA) were administered to evaluate global cognitive function. FOGQ was applied to PD subjects to assess the severity of FOG. The FOGQ was a 6-item questionnaire comprising 2 questions evaluating the overall gait performance and 4 questions of FOG severity. Each item was scored 0–4 and greater score reflected more severe FOG. All the assessments were carried out in the practically defined OFF state (at least 12 h of dopaminergic replacement therapy withdrawal).

### Imaging acquisition

MRI scans were carried out by a 3T SIEMENS MAGNETOM Prisma scanner (Siemens Healthineers, Erlangen, Germany) with a 64-channel head coil. Identical parameters were used to obtain structural 3D T1-weighted images and resting state fMRI data among participants.

Structural 3D T1-weighted images were collected using magnetization-prepared rapid gradient echo (MP-RAGE) sequence (repetition time = 2300 ms, echo time = 2.26 ms, inversion time = 900 ms, flip angle = 8°, field of view = 256 mm × 256 mm, matrix = 256 × 256, number of slices = 186, and voxel size = 1 mm × 1 mm × 1 mm with no gap). Resting state functional images were obtained by an echo-planar imaging sequence (repetition time = 750 ms, echo time = 30 ms, number of slices = 64, flip angle = 54°, field of view = 222 mm × 222 mm, matrix size = 74 × 74, and voxel size = 3 mm × 3 mm × 3 mm with no gap). Resting state data were acquired by a scanning run of 6 min and 11 s. During resting-state fMRI scanning, subjects were under the instruction of keeping motionless, closing their eyes and not thinking about anything in particular, which was later affirmed by post-scan debriefing. Scanner noise was reduced by headphones, while foam padding was applied to lessen head motion. Additionally, PD patients were scanned in OFF dopaminergic medication state (after overnight washout for more than 12 h). The dynamics were under close monitoring during each fMRI scan and a repeated scan would be performed if the image had been corrupted by excessive head motion.

### Gray matter density

Gray matter density (GMD) was calculated with FSL voxel based morphometry (VBM) (FSL version 6.0). First, brain-extracted images were segmented into gray matter (GM), white matter, and cerebrospinal fluid (CSF) tissue maps. Then, we created a study-specific gray matter template in two steps. In a first run, the GM images were affine-registered to the ICBM-152 gray matter template and the obtained images were averaged to create a first-pass template. In a second run, the gray matter images were non-linearly registered to the first-pass template and subsequently averaged to attain the final template at 2 mm × 2 mm × 2 mm resolution in standard space. Finally, the gray matter images were non-linearly registered to the final template and smoothed with an 8 mm Gaussian kernel filter. The voxel values ranged between 0 and 1, representing the percentage of a voxel being gray matter tissue. The voxel-wise values were averaged in the left and right visual brain area from automated anatomical labeling 90 (AAL90) atlas. The visual brain areas include calcarine fissure and surrounding cortex, cuneus, lingual gyrus, superior occipital lobe, middle occipital lobe and inferior occipital lobe. The weighted averages of the regions were calculated, with voxels contributing to the average of a region based on their probability of being part of that region.

### Quality control

Image quality control was carried out prior to data preprocessing. Mean framewise displacement and maximum displacement were calculated. The mean framewise displacement was determined in accord with the formula of Power et al. (2012), which combined translational (*x*, *y*, *z* axes) and rotational (pitch, yaw, roll) scan-to-scan displacement using six parameters obtained for all the participants in the realignment steps. The maximum displacement refers to the maximum absolute translation of each volume when compared to the reference volume in the *x*, *y*, and *z* directions.

Acquisitions were excluded if the mean framewise displacement values > 0.2 mm or if the maximum displacement was greater than one voxel size. Furthermore, we regressed out nuisance signals including 24 motion parameters, as well as signals from white matter and CSF voxels by using Resting-State fMRI Data Analysis Toolkit V1.24 (RESTplus V1.24).

### Preprocessing of functional magnetic resonance imaging data

Data preprocessing was carried out using RESTplus V1.24 based on SPM12 software^1^ implemented in MATLAB (version R2013b, MathWorks, Inc., Natick, MA, United States). To adjust interscan head motions, we realigned data to the first volume and subsequently segmented them into gray matter, white matter and CSF by using the Tissue Probability Map template. The data were smoothed with a 6 mm full width half maximum Gaussian kernel following normalization to a standard brain template in Montreal Neurological Institute space.

### Functional connectivity

A seed reference time course was obtained by averaging the time courses within each region of interest (ROI). Correlation analysis was carried out in a seed-to-seed approach. The identified regions within VN with significantly different GMD in PD-FOG were combined together and then used as the seed. Other FOG-related brain areas in AAL90 including frontal lobe (inferior frontal gyrus, middle frontal gyrus, superior frontal gyrus, gyrus rectus, precentral gyrus, supplementary motor area, paracentral lobule), midbrain, basal ganglia (caudate nucleus, putamen, pallidum) and thalamus were chosen according to previous studies (Bharti et al., 2019) and used as the other seed in this step (Tessitore et al., 2012; Fling et al., 2013). Each correlation coefficients were further transformed to *z*-values using the Fisher r-to-z transformation. All these analyses were completed within RESTplus.

### Statistical analysis

All the statistical analyses were performed in SPSS 26.0.0.0 (SPSS, Inc., Chicago, IL, United States). Demographic and clinical characteristics data were compared between groups using ANOVA models.

To examine the structural differences within VN, two sample *t*-tests were applied to compare the difference of GMD and FC between groups when the data were normally distributed, and the Mann–Whitney test was used if the data were not.

To examine the differences of GMD and resting-state FCs, the general linear model analyses were performed. The dependent variables were GMD or FCs, the model factor was group (i.e., PD-FOG or PD-NFOG, and HC). All the models were adjusted for age and sex, and the false discovery rate (FDR) correction was used.

To explore the relationship between GMD, FC and FOG severity, partial correlation was applied to study the correlation between FOGQ score, GMD and FC. *P*-value < 0.05 after FDR correction was considered significant.

## Results

### Demographic and clinical characteristics

Seventy-eight PD patients (25 with FOG and 53 without FOG) and twenty-nine HCs were included in the final analysis after qualified for image quality and motion control. The demographic and clinical characteristics of these participants are summarized in Table 1. The age of HC group was significantly lower than PD-FOG and PD-NFOG groups (*p* < 0.0001). Accordingly, within the analysis of GMD and FC, a general linear model including age and sex as covariates was applied to regress out the effect of unmatched age between groups when comparing PD subjects and HCs. There were no significant differences between the PD-FOG and PD-NFOG groups in terms of age, gender, disease duration, UPDRS-III, LEDD, MMSE, MoCA, and HAMD scores (Table 1).

**TABLE 1:** Demographic and clinical characteristics of HCs and PD patients with and without FOG.

	HCs (*n* = 29)	PD-FOG (*n* = 25)	PD-NFOG (*n* = 53)	*P-value* (FOG vs. NFOG)
Age, mean (SD)	52.31 (7.30)	64.48 (13.10)	62.36 (8.29)	0.387
Sex (male/female)	13/16	11/14	28/35	0.628
Disease duration, year	–	10.10 (4.42)	8.25 (3.81)	0.063
Age of onset, mean (SD)	–	54.38 (12.33)	54.33 (9.62)	0.985
UPDRS-III score, mean (SD)	–	45.74 (15.07)	38.48 (14.10)	0.059
LEDD, mean (SD), mg/day	–	1093.75 (473.57)	926.93 (342.74)	0.081
MMSE score, mean (SD)	–	25.60 (3.51)	26.02 (3.59)	0.632
MoCA score, mean (SD)	–	22.32 (5.11)	21.98 (4.65)	0.774
HAMD score, mean (SD)	–	12.31 (5.98)	9.24 (8.35)	0.225

HC, healthy control; PD, Parkinson’s disease; FOG, freezing of gait; UPDRS, Unified Parkinson’s Disease Rating Scale; LEDD, levodopa dose; MMSE, Mini-Mental State Examination; MoCA, Montreal Cognitive Assessment; HAMD, Hamilton Rating Scale of Depression.

### Structural change of visual cortex

**FIGURE 1:**
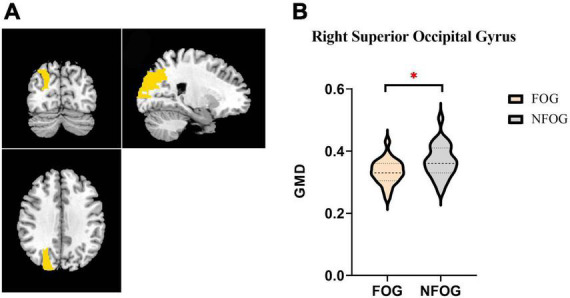
Significant intergroup differences in superior occipital gyrus. Panel **(A)** shows coronal, sagittal, and horizontal plane of the brain. Right superior occipital gyrus is marked yellow. Panel **(B)** demonstrates the gray matter density of right superior occipital gyrus in PD-FOG and PD-NFOG patients. Significant decline was presented in PD-FOG. GMD, gray matter density; FOG, freezing of gait; NFOG, non-freezing of gait. *p < 0.05.

It was observed that the GMD of right superior occipital gyrus significantly decreased in PD-FOG patients compared to PD-NFOG patients (PD-FOG vs. PD-NFOG: 0.33 ± 0.04 vs. 0.37 ± 0.05, *p* = 0.005) (Figure 1). The GMD of right superior occipital gyrus was also significantly decreased in PD-FOG compared to HCs. All the significance was independent from age and sex (PD-FOG vs. HCs: 0.33 ± 0.04 vs. 0.39 ± 0.06, *p* = 0.002). There was no significant difference of GMD between PD-NFOG and HC (PD-NFOG vs. HCs: 0.37 ± 0.05 vs. 0.39 ± 0.06, *p* = 0.110). No significant correlation was detected between GMD of right superior occipital lobe and FOGQ score (*r* = 0.032, *p* = 0.878).

### Functional connectivity between visual network and other freezing of gait-related region of interests

**FIGURE 2:**
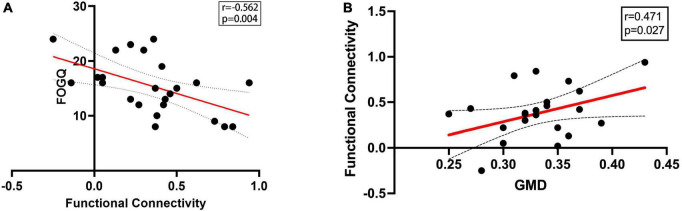
Correlation between FOGQ score, GMD and functional connectivity. **(A)** The functional connectivity between right superior occipital gyrus and right paracentral lobule was negatively associated with FOGQ score. **(B)** The functional connectivity between right superior occipital gyrus and right paracentral lobule was positively correlated with GMD of right superior occipital gyrus in PD-FOG. GMD, gray matter density; FOGQ, freezing of gait questionnaire.

Functional connectivity was calculated between right superior occipital gyrus and previously reported FOG-related ROIs as described in Methods. Functional connectivity between right superior occipital gyrus and right paracentral lobule significantly decreased in PD-FOG compared to PD-NFOG (*p* = 0.045). The identified seed pairing with group differences in functional connectivity strength was then analyzed for correlation with clinical metrics of freezing. The connectivity strength between right superior occipital gyrus and right paracentral lobule was negatively associated with FOGQ score (*r* = -0.562, *p* = 0.004) (Figure 2A).

### The relationship between functional connectivity and gray matter density

Pearson correlation analysis revealed significant correlations between FC and GMD in PD-FOG. The FC between right superior occipital gyrus and right paracentral lobule was detected to be positively correlated with GMD of right superior occipital gyrus (*r* = 0.471, *p* = 0.027) (Figure 2B). However, the significant correlation between FC and GMD was failed to be detected in PD-NFOG group (*r* = 0.152, *p* = 0.288).

## Discussion

In this study, we observed that compared to PD-NFOG and HCs, the gray matter density in right superior occipital gyrus was significantly lower in PD-FOG. The functional connectivity between right superior occipital gyrus and right paracentral lobule in PD-FOG was decreased compared to PD-NFOG and HCs. Moreover, the connectivity strength was negatively associated with FOGQ score and positively correlated to gray matter density. These results revealed that the structural change of VN and functional alterations between visual and motor networks potentially underlie the mechanism of FOG.

The occipital lobe, consisting of primary visual cortex and visual association cortex, is the core of visual processing. The superior occipital gyrus is one of the key regions in the visual association cortex responsible for higher level visual association processing and interpreting visual images (Alves et al., 2012). A recent video-guided motor imagery study found that PD-FOG showed increased activity in the superior occipital gyrus than PD-NFOG when watching the video clips of FOG turning (Huang et al., 2021). The finding implied that superior occipital gyrus might occupy a special role in enhancing spatiotemporal demands in gait generating. The decrease of right superior occipital gyrus density may serve as neuroanatomical basis of impaired visuomotor function which might interfere with the visual-motor integrating process pertaining to freezing of gait.

Based upon the findings of structural characteristics of the brain in PD-FOG, we observed a decreased functional connectivity between right superior occipital gyrus and right paracentral lobule that is related to the control of motor innervations in lower extremity. Moreover, such FC is closely related to the GMD of this region. Converging evidence has supported that resting state functional connectivity within the visual network decreased in PD-FOG patients (Tessitore et al., 2012; Canu et al., 2015). Moreover, Zhou et al. (2019) observed significant decrease of the metabolism of the primary visual cortex using PET-CT in PD-FOG group compared with PD-NFOG (Zhou et al., 2019). It has been postulated that hypoactivation in visual brain area might underlie the deficits of visual network related to gait disturbances.

The lower connectivity strength in the resting state may explain hyperactivation of visual network in motor tasks reported in previous studies. Gilat et al. (2015) showed that PD-FOG patients required more activation in visual and motor cortices to initiate walking in VR task. A task functional MRI study also detected that PD-FOG patients showed higher activation in the parieto-occipital areas and lower activation in the basal ganglia during feet movement tasks (Piramide et al., 2020). Another task fMRI study showed that processing of an indirect walk cue in a VR paradigm was paralleled by significantly increased BOLD signal in the extra-striate visual cortex (Shine et al., 2013). The findings were corroborated by the observation that superior occipital lobule as well as the precentral and postcentral gyrus exhibited greater activation in PD-FOG (Huang et al., 2021). The enhanced activation detected in VN in the above studies was contrary to our findings that functional connectivity of VN were decreased in the resting state. The authors interpreted their results as indicating that the hyperactivation of visual-motor circuit might act to compensate the inadequate function in the resting state in gait disturbance of PD-FOG. The selected ROIs in the current study were FOG-related brain regions reported in previous studies (Bharti et al., 2019; Song et al., 2021). It would be meaningful to explore how other brain regions interact with visual network in FOG in future studies.

The contrasting shifts of visual network activation between resting state and tasks are probably associated with different motor phase of PD-FOG patients. It is worth noting that visual network involves in normal gait in healthy subjects. A SPECT study revealed that visual cortex activated in healthy subjects during voluntary walking (Fukuyama et al., 1997) which indicated visual input played a crucial role in locomotion. The hypoactivation of visual-motor circuit in FOG resting state possibly represents deficient interaction between vision and locomotion network. This may contribute to insufficient preparation for gait initiation hence manifested as FOG. To generate steps, greater activation of visual-motor circuitry will be required during motor preparation or movement onset to compensate primary decrease of visual-motor connectivity in resting state. All the MRIs in our study were obtained in medication-off state (all anti-parkinsonian drugs withdrawal) to exclude the potential influence from the medication on the characteristics of the brain in fMRI scanning. Dopaminergic therapy changes the brain activity (Maillet et al., 2015). Future study could investigate the influence of medication on visual network in FOG by comparing on- and off-medication state functional activity.

We also observed a significant association between the alterations in structural and in functional characteristics in FOG. The functional connectivity between right superior occipital gyrus and right paracentral lobule was positively correlated with gray matter density of right superior occipital gyrus. This finding implied a interaction between FOG-related structural and functional change of the visual network. The loss of gray matter in visual network might serve as the underlying anatomical basis that reorganize the functional gradient of visuomotor network. It is equally possible that the impaired locomotion network disturbed visual structure. It is worth noting that the significant structure-function correlation was detected in PD-FOG but not in PD-NFOG. We assume that visual network change might be FOG-specific deficit.

The present study confirmed clinical association of functional change in visual network with FOG. Our data revealed inverse relationship between FOGQ score and resting state connectivity suggesting worse vision-locomotion coordination in patients with more severe FOG. This is coincided with a paired-pulse transcranial magnetic stimulation (TMS) study. The study demonstrated an exaggerated inhibitory response of the right primary motor cortex (M1) to inputs traveling from primary visual area at given interstimulus intervals in PD-FOG with higher FOGQ score. This finding indicated inadequate M1 facilitation in response to visual information inputs in movement (Strigaro et al., 2020). TMS treatment targeting right superior occipital gyrus and right paracentral lobule might also be applied to verify whether strengthening of visuomotor connection lead to alleviation of FOG. Although visuomotor connectivity appeared to be associated with disease severity, the gray matter loss was not correlated with FOGQ score. This suggested that visual brain network structural damage might be constant in the disease course.

Several limitations should be noted. First, healthy controls were younger than PD participants in our study. Although we adjusted for age, future study are still needed to control the variables of basic characteristics to make sure groups are well-matched. Second, FOG appears in waves and is unexpected, often not showing up during examinations. We partly relied on our patients’ self-reports of FOG, which could be skewed by recollection bias. More objective assessment tools and effective FOG-provoking tasks are required to fully evaluate FOG. Third, there were certain difference in the sample size between PD-FOG group and PD-NFOG group, which might affect precision of the results. Thus our findings warrant confirmation on balanced grouping design.

Nevertheless, our study provides further insights into the dysfunctional mechanisms of gait that visual network deficiency might underlie the mechanism of FOG in Parkinson’s disease, suggesting targeting visuomotor network may be potentially developed into a new neuromodulation strategy for future FOG treatment.

## Data availability statement

The raw data supporting the conclusions of this article will be made available by the authors, without undue reservation.

## Ethics statement

The studies involving human participants were reviewed and approved by the Medical Ethical Review Committee of Beijing Tiantan Hospital. The patients/participants provided their written informed consent to participate in this study.

## Author contributions

LG and DS contributed to the organization and execution of the research project. RY and DS drafted the manuscript. LG, RY, DS, ZL, ZW, XW, HM, and YB contributed to the acquisition, post-processing, and analysis of the data. TF, JZ, and GM revised the manuscript. All authors contributed to the article and approved the submitted version.

## Footnotes

1
http://www.fil.ion.ucl.ac.uk/spm/
